# One-Step Synthesis of Heterostructured Mo@MoO_2_ Nanosheets for High-Performance Supercapacitors with Long Cycling Life and High Rate Capability

**DOI:** 10.3390/nano14171404

**Published:** 2024-08-28

**Authors:** Ao Cheng, Yan Shen, Tao Cui, Zhe Liu, Yu Lin, Runze Zhan, Shuai Tang, Yu Zhang, Huanjun Chen, Shaozhi Deng

**Affiliations:** State Key Laboratory of Optoelectronic Materials and Technologies, Guangdong Province Key Laboratory of Display Material and Technology, School of Electronics and Information Technology, Sun Yat-sen University, Guangzhou 510275, China; chengao@mail2.sysu.edu.cn (A.C.); cuit7@mail2.sysu.edu.cn (T.C.); liuzh336@mail2.sysu.edu.cn (Z.L.); liny398@mail2.sysu.edu.cn (Y.L.); zhanrz3@mail.sysu.edu.cn (R.Z.); tangsh58@mail.sysu.edu.cn (S.T.); stszhyu@mail.sysu.edu.cn (Y.Z.); chenhj8@mail.sysu.edu.cn (H.C.); stsdsz@mail.sysu.edu.cn (S.D.)

**Keywords:** supercapacitor, Mo@MoO_2_ nanosheets, one-step synthesis, core-shell structure, heterogenous interface

## Abstract

Supercapacitors have gained increased attention in recent years due to their significant role in energy storage devices; their impact largely depends on the electrode material. The diversity of energy storage mechanisms means that various electrode materials can provide unique benefits for specific applications, highlighting the growing trend towards nanocomposite electrodes. Typically, these nanocomposite electrodes combine pseudocapacitive materials with carbon-based materials to form heterogeneous structural composites, often requiring complex multi-step preparation processes. This study introduces a straightforward approach to fabricate a non-carbon-based Mo@MoO_2_ nanosheet composite electrode using a one-step thermal evaporating vapor deposition (TEVD) method. This novel electrode features Mo at the core and MoO_2_ as the shell and demonstrates exceptional electrochemical performance. Specifically, at a current density of 1 A g^−1^, it achieves a storage capacity of 205.1 F g^−1^, maintaining virtually unchanged capacity after 10,000 charge–discharge cycles at 2 A g^−1^. The outstanding long-cycle stability is ascribed to the vertical two-dimensional geometry, the superior conductivity, and pseudocapacitance of the Mo@MoO_2_ core-shell nanosheets. These attributes significantly improve the electrode’s charge storage capacity, charge transfer speed, and structural integrity during the cycling process. The development of the one-step grown Mo@MoO_2_ nanosheets offers a promising way for the advancement of high-performance, non-carbon-based supercapacitor nanocomposite electrodes.

## 1. Introduction

As environmental pollution becomes an increasingly pressing concern, the urgency to cultivate an eco-friendly society intensifies. Concurrently, there is a growing necessity to explore innovative, renewable, and clean energy alternatives [1,2,3,4]. While a variety of innovative energy storage methods, such as lithium-ion batteries, lithium–sulfur batteries, and solar energy are available, supercapacitors distinguish themselves by offering high power density, fast charging and discharging capabilities, and low cost. These advantages position supercapacitors as having substantial developmental potential in the quest for efficient energy solutions [5,6,7,8,9,10]. The performance of supercapacitors is directly influenced by their electrode materials, which are crucial components in determining the device’s overall efficiency [11]. Supercapacitor electrode materials, due to their varied energy storage mechanisms, are categorized into two main types: electric double-layer capacitors and Faradaic pseudocapacitor materials [12,13]. Electric double-layer capacitor materials store energy through the surface adsorption of ions with stability, while Faradaic pseudocapacitor materials utilize redox reactions, enabling them to achieve higher storage capacities [14,15,16,17,18]. Consequently, current research is concentrating on integrating these energy storage mechanisms to simultaneously attain high capacity and excellent cyclic stability. This has led to the development of composite electrodes that combine metal oxides or metal sulfides with carbon materials, among others, showcasing the innovative direction of supercapacitor advancement [19,20,21,22,23]. However, the electrodes previously discussed are predominantly heterostructures composed of pseudocapacitive materials and double-layer carbon-based materials. The preparation process for these electrodes is comparatively complex, presenting challenges in their fabrication. Li et al. combined the template method and chemical vapor deposition (undergoing freeze-drying, carbonization at 500 °C for 1 h, and deionized water washing in three steps) to obtain MnO@HCNb and NaNbO_3_@HCNb (hollow carbon nanobox) nanocomposite electrodes [24]. Ma et al. first synthesized WO_3-*x*_-N14-H8 (~14 mL NH_4_Ac, ~8 mL H_2_O_2_) using the hydrothermal method, and then after annealing at 500 °C for 10 h, they finally obtained a WO_3-*x*_-N14-H8/CC (carbon cloth) composite electrode [25]. Dai et al. first synthesized MoS_2_ via a hydrothermal method, then obtained MoS_2_@PANI (polyaniline) by stirring, and finally achieved a MoS_2_@C composite electrode after annealing at 600 °C for 2 h [26]. The methodologies described typically necessitate two or more processing stages, resulting in a more complicated preparation process, lower efficiency, and higher costs for the electrode materials. Therefore, the development of a method capable of synthesizing high-performance heterostructured nanocomposite electrodes in one step has considerable significance for the broader application of supercapacitors.

MoO_2_ is recognized for its excellent electrical conductivity, variable valence states, high electrochemical activity, and high theoretical specific capacitance. These attributes make it a promising candidate for use as a pseudocapacitive electrode material in energy storage applications. Nanocomposite electrodes that incorporate MoO_2_ and electric double-layer carbon nanomaterials have garnered widespread interest due to their synergistic properties, enhancing both capacitance and energy storage capabilities [27,28,29,30,31]. The MoO_2_@CF (carbon fiber) nanomaterials were synthesized by hydrothermal method, achieving a storage capacity of 405.4 F g^−1^ at a current density of 2 A g^−1^, maintaining 64.5% capacity after 2500 cycles [27]. The MoO_2_/CF (carbon fiber) electrode was fabricated by electrochemical deposition, showing a capacity of 315 F g^−1^ at 5 A g^−1^, and still exhibited a good capacity retention rate of 92.3% after 5000 cycles [32]. The MoO_2_/Gr (graphene) electrode was synthesized through a templating method, achieving an ultra-high capacitance of 1212 F g^−1^ [33]. Nonetheless, the fabrication of these electrodes invariably involves multi-step synthesis processes and additional carbonization treatments, adding complexity and potentially increasing the overall production costs.

In this research, we present the first direct synthesis of vertically oriented, non-carbon-based Mo@MoO_2_ core-shell structured nanosheets as an electrode, achieved through a novel one-step thermal evaporating vapor deposition (TEVD) method. This approach obtains a composite electrode with a unique heterogeneous interface structure, where the outer MoO_2_ uniformly coats the inner Mo nanosheets, both exhibiting desirable monocrystalline properties. Electrochemical tests reveal that at a current density of 1 A g^−1^ the electrode achieves a storage capacity of 205.1 F g^−1^. Remarkably, after 10,000 cycles at a current density of 2 A g^−1^, the capacitance shows minimal degradation, maintaining retention of 99.9%. Even at an elevated current density of 10 A g^−1^, the capacity retention rate remains high at 76.2%. The energy storage performance of the Mo@MoO_2_ nanosheets is largely due to their vertical two-dimensional geometry and distinct core-shell architecture. These results underline the potential of the Mo@MoO_2_ nanosheet heterostructure as a high-performance electrode material for supercapacitors. Furthermore, the proposed one-step synthesis method provides the way for the development of a new class of non-carbon nanocomposite electrode materials.

## 2. Materials and Methods

### 2.1. Preparation

The heterostructured Mo@MoO_2_ nanosheets were fabricated using a custom-built thermal evaporating vapor deposition (TEVD) apparatus, as depicted in the structural schematic (see Figure S1). The source material for thermal evaporation consisted of a high-purity metal Mo boat (>99.95% purity, Luoyang Zhixing Trading Co., Ltd., Luoyang, China). The Mo boat was meticulously cleaned through ultrasonic washing with acetone followed by ethanol. Subsequently, it was inverted and firmly mounted on two copper electrodes. Ni foam, with a thickness of approximately 1 mm (Hefei Kojing Material Technology Co., Ltd., Hefei, China), served as the substrate. The Ni foam was first ultrasonically cleaned for 10 min in a 2 mol L^−1^ H_2_SO_4_ solution to remove the oxide layers. This step was followed by subsequent ultrasonic washings with acetone and ethanol, respectively, ensuring the substrate was thoroughly cleaned and ready for use in the synthesis process. Following vacuum drying, the Ni substrate was weighed and recorded as *m*_1_. The substrate was then placed directly below the Mo boat on a copper platform. The TEVD chamber was then evacuated to 5 Pa using a mechanical pump. After achieving this vacuum level, Ar (200 sccm) and H_2_ (100 sccm) gases were introduced into the system. The H_2_ here can inhibit the oxidation of the nickel foam substrate. The heating process commenced once the vacuum stabilized at 280 Pa. By energizing the two copper electrodes, the temperature was ramped up to 1350 °C at a rate of 100 °C/min. Subsequently, the flow of H_2_ gas was stopped, and the system was maintained at 78 Pa for 1 h. The Mo boat fully reacted with the residual oxygen in the chamber, forming MoO_2_ vapor. This vapor then underwent a disproportionation reaction to form elemental Mo, which deposited on the Ni substrate to form Mo nanosheets. The surface of the Ni substrate was almost completely covered by Mo nanosheets, preventing oxygen from contacting the Ni substrate and thereby inhibiting the oxidation of the Ni foam. The surface of the Mo nanosheets continued to react with the residual oxygen, forming a MoO_2_ shell around them. The thickness and content of the MoO_2_ layer can be controlled by adjusting the cooling rate during the cooling phase of TEVD. In this experiment, a rapid cooling rate (600 °C min^−1^) was used to obtain a relatively thin MoO_2_ layer. Finally, the system was allowed to cool back to room temperature, at which point the sample was obtained. The sample was weighed and recorded as *m*_2_, and the mass of Mo@MoO_2_ nanosheets was calculated by the difference method using *m* = *m*_2_ − *m*_1_.

### 2.2. Characterization

The surface morphology of the Mo@MoO_2_ nanosheets was examined using scanning electron microscopy (SEM, Supra 60, Zeiss, Jena, Germany). To delve deeper into the crystal structure, a transmission electron microscope (TEM, Titan3 G2 60–300, manufactured by FEI Electron Optics B.V., Hillsboro, OR, USA) was utilized. The elemental composition, distribution, and ratio of the samples were studied using the energy dispersive spectrometer (EDS) integrated within the TEM system. X-ray diffraction (XRD, D-max 2200 VPC, Rigaku, Tokyo, Japan) was employed to characterize the crystal phase of the samples. The component mass ratios of Mo and MoO_2_ were analyzed through thermogravimetric analysis (TGA, TG209F1, Netzsch, Selb, Germany). Additionally, the composition and valence states of the elements in the samples were thoroughly investigated using X-ray photoelectron spectroscopy (XPS, model Escalab 250Xi, provided by Thermo Fisher Scientific, Waltham, MA, USA).

### 2.3. Electrochemical Measurements

The electrochemical performance of the Mo@MoO_2_ nanosheets was evaluated using a three-electrode system, where the nanosheets served as the working electrode. A platinum sheet was used as the counter electrode, and a saturated calomel electrode functioned as the reference. The electrolyte was a 2 mol L^−1^ KOH solution. Various electrochemical techniques were employed for comprehensive testing, including electrochemical impedance spectroscopy (EIS), cyclic voltammetry (CV) at different scan rates ranging from 10 mV s^−1^ to 100 mV s^−1^, and galvanostatic charge–discharge (GCD) measurements under varying current densities from 1 A g^−1^ to 10 A g^−1^. Additionally, long-cycle stability tests were conducted at 2 A g^−1^. These assessments were carried out on a commercial electrochemical workstation (CHI660E, Shanghai Chenhua, Shanghai, China) with the voltage window ranging between 0.2 V and 0.7 V. Two samples of the Mo@MoO_2_ nanosheets with similar mass were selected as symmetric electrodes with cellulose as the separator. The separator was fully saturated with 2 mol L^−1^ KOH electrolyte and then assembled into a coin-type symmetrical supercapacitor (CR2032, Hefei Kojing Material Technology Co., Ltd., Hefei, China) to further explore its energy storage performance.

## 3. Results and Discussion

### 3.1. Synthesis Process and Structural Characterization

**Figure 1:**
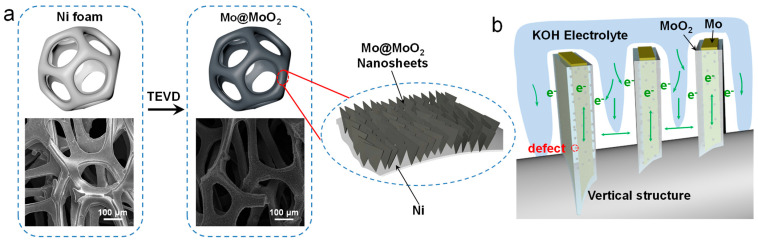
(**a**) Typical SEM images of the Ni foam framework substrate before and after the TEVD synthesis of Mo@MoO_2_ core-shell heterostructured nanosheets, and the corresponding schematic diagrams. (**b**) Depiction of ion/electron transport behavior during the charge–discharge process.

The thermal evaporating vapor deposition (TEVD) one-step synthesis process is depicted in Figure 1a, which shows the formation of Mo@MoO_2_ core-shell heterostructured nanosheets utilizing a three-dimensional Ni foam framework as the current collector. The nanosheets comprehensively cover the Ni substrate and exhibit vertical growth from the substrate surface. The core-shell structure is characterized by an inner layer of metallic Mo nanosheets, which originates from the disproportionation decomposition and deposition of MoO_2_ vapor. Concurrently, the outer layer comprises a MoO_2_ coating that forms as the Mo undergoes oxidation during the cooling stage. Remarkably, the development of this complex core-shell nanoarchitecture necessitates only a one-step heating and cooling cycle. The chemical reactions are as follows [34,35]:

MoO_2_ (*v*) → MoO_3_ (*v*) + Mo (*s*) (1)

Mo (*s*) + O_2_ (*v*) → MoO_2_ (*s*) (2)

Figure 1b illustrates the heterostructured Mo@MoO_2_ nanosheets, which are vertically aligned and exhibit the high specific surface area characteristic of two-dimensional materials, a feature that significantly improves electrolyte infiltration. The interface of the heterostructure is marked by an abundance of defects, which play a crucial role in providing numerous active sites and channels for ion/electron storage and transport. Importantly, the inner layer of the Mo nanosheets demonstrates exceptional electrical conductivity, serving as a transitional layer that not only facilitates electron transfer between the outer MoO_2_ layer and the current collector, but also maintains the structural integrity of the electrode material during the cyclic charging–discharging. The outer MoO_2_ layer, on the other hand, is instrumental in contributing to the electrode’s pseudocapacitive properties, thereby substantially increasing the specific capacitance of the nanocomposite electrode. This synergistic relationship between the core and shell materials enhances both the energy storage capacity and the durability of the electrode.

**Figure 2:**
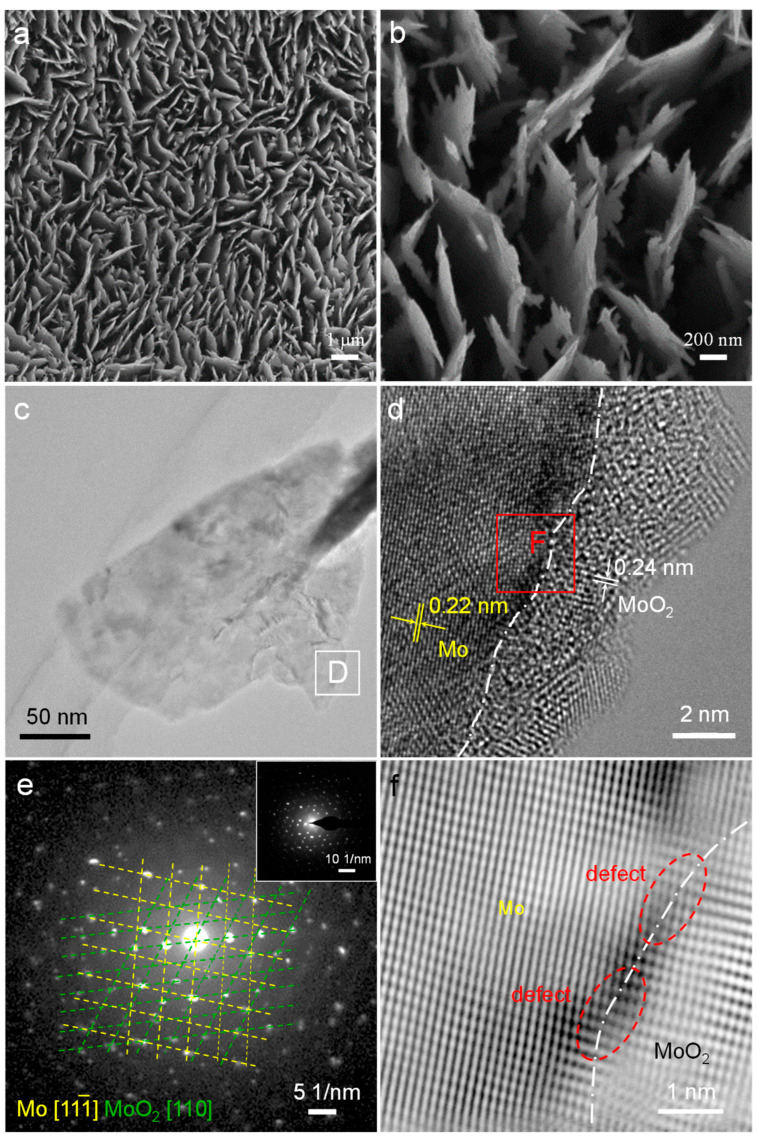
Micro-morphologies and structure of the Mo@MoO_2_ nanosheets. (**a**,**b**) SEM images at different magnifications. (**c**) Low-magnification TEM image. (**d**) HRTEM image of the marked area D in (**c**). (**e**) The SEAD pattern of the nanosheet in (**c**). (**f**) The Inverse Fourier Transform result of the marked area F in (**d**).

The microstructure, morphology, and elemental composition of the Mo@MoO_2_ nanosheets were comprehensively analyzed using SEM, TEM, and EDS. SEM images at various magnifications, shown in Figure 2a,b, reveal that the nanosheets grow vertically relative to the Ni substrate and uniformly envelop the substrate’s surface. The absence of impurities on the surface of these nanosheets was confirmed through elemental analysis of individual sheets, as illustrated in Figure S2. Typically, the nanosheets have a width of 600~700 nm, a thickness of 10~15 nm, and are separated by gaps of approximately 200 nm (Figure 2b). This vertical orientation and the large specific surface area of the flake-like structures enhance the contact area with the electrolyte, which is advantageous for the electrochemical performance of the material. Further TEM observations of the Mo@MoO_2_ nanosheets, depicted in Figure 2c, reveal that the nanosheets possess fan-shaped structures with narrower bottoms and wider tops, aligning with the SEM findings. Figure 2d presents an enlarged image of the D area at the edge of Figure 2c, which shows a HRTEM image that clearly delineates the heterointerface between Mo and MoO_2_. The lattice spacing of the Mo in the inner layer is identified as 0.22 nm, corresponding to the (110) plane of body-centered cubic (bcc) Mo. Simultaneously, the MoO_2_ in the outer layer exhibits a lattice spacing of 0.24 nm, which matches with the ($\bar{2}11$) plane of MoO_2_. The thickness of the MoO_2_ outer shell is determined to be approximately 3~4 nm, a result corroborated by EDS mapping analysis (Figure S2). This detailed characterization underlines the intricate nanoarchitecture of the Mo@MoO_2_ nanosheets, highlighting their potential for enhancing the performance of supercapacitors through improved electrolyte accessibility and increased active surface area for electrochemical reactions.

The detailed structure and phase composition of the Mo@MoO_2_ nanosheets were further elucidated through selected-area electron diffraction (SAED), providing insightful analysis into their crystallinity. The SAED pattern of an individual Mo@MoO_2_ nanosheet, as shown in Figure 2e, displays two distinct sets of diffraction spots. These are delineated by yellow and green dotted lines, which correspond to the body-centered cubic (bcc) phase of Mo and the monoclinic phase of MoO_2_, respectively. This observation unequivocally confirms that both Mo and MoO_2_ within the Mo@MoO_2_ composite material exist in their monocrystalline forms. The meticulous investigation of the heterointerface between the inner Mo and outer MoO_2_ layers through Inverse Fourier Transform analysis, as demonstrated in Figure 2f, uncovers a significant number of atomic arrangement defects. These defects, highlighted by red dashed circles, are situated at the critical juncture where the metallic Mo core meets the MoO_2_ shell. Such imperfections are pivotal for the electrochemical performance of the Mo@MoO_2_ nanosheets, as they likely enhance the electrode’s ability to store electrons and ions.

**Figure 3:**
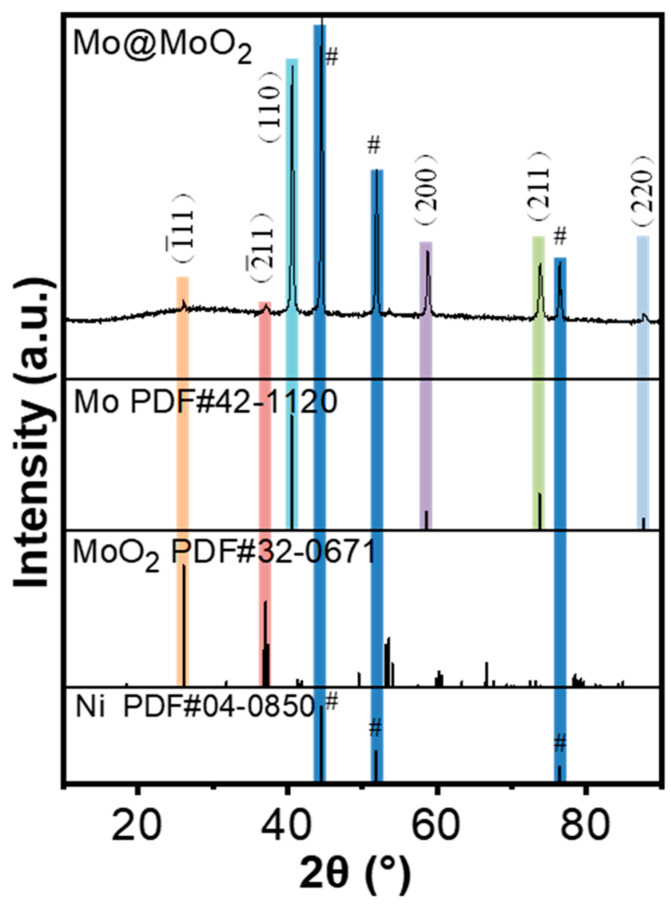
XRD characterization of the prepared Mo@MoO_2_ nanosheets (compared with Mo, MoO_2_ and Ni standard PDF phase cards, respectively).

Figure 3 presents the X-ray diffraction spectroscopy (XRD) analysis of the Mo@MoO_2_ nanosheets, revealing their crystal structure. The XRD pattern shows prominent diffraction peaks at 2θ values of 40.5°, 58.6°, 73.5°, and 87.6°, which are attributed to the (110), (200), (210), and (200) crystal planes of the Mo phase, respectively. These peaks align perfectly with the standard card of Mo (JCPDS PDF#42-1120). Additionally, there are weaker diffraction peaks at 2θ = 26° and 37°, corresponding to the ($\bar{1}$11) and ($\bar{2}11$) crystal planes of MoO_2_, respectively, which match the standard card of MoO_2_ (JCPDS PDF#32-0671). The sharpness and strength of the Mo characteristic peaks suggest a high degree of crystallization. Besides the identified peaks for Mo and MoO_2_, peaks marked with the # symbol correspond to the foam Ni substrate (JCPDS: 04-0850). The absence of any extraneous diffraction peaks confirms the purity of the synthesized nanocomposite, comprising solely Mo and MoO_2_ phases. This analysis not only validates the successful synthesis of the Mo@MoO_2_ nanocomposite but also emphasizes its high crystallinity and purity.

### 3.2. Component Analysis

**Figure 4:**
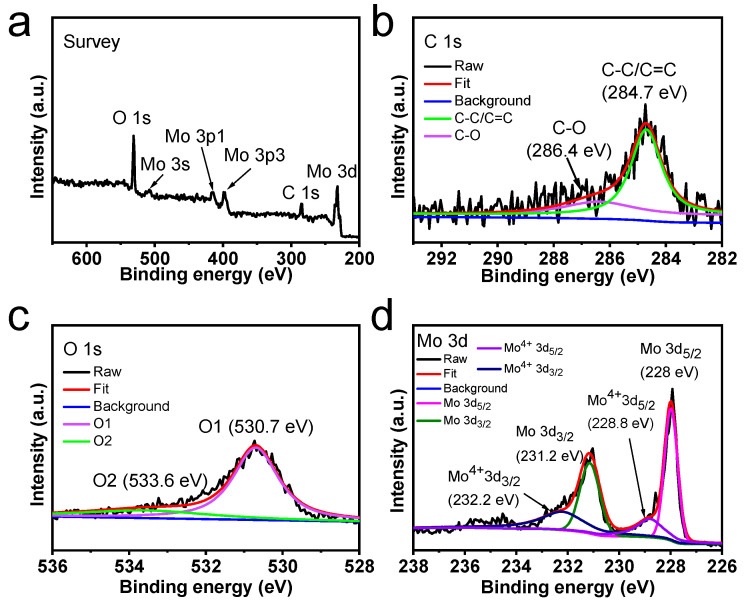
XPS analysis of the prepared Mo@MoO_2_ nanosheets. (**a**) Wide-scanning survey XPS spectrum. (**b**–**d**) High-resolution XPS spectra of C 1s, O 1s, and Mo 3d in Mo@MoO_2_, respectively.

The X-ray photoelectron spectroscopy (XPS) analysis provided a detailed insight into the elemental composition and chemical states of the Mo@MoO_2_ nanosheets. As depicted in Figure 4a, the full-spectrum XPS analysis confirms the presence of Mo, O, and C elements within the nanosheets. This elemental composition is crucial for understanding the material’s structure and potential functionalities. A significant peak in the high-resolution C 1s spectrum, observed at 284.7 eV in Figure 4b, is attributed to C-C/C=C bonds, while a weak shoulder peak at 286.4 eV corresponds to C-O bonds. Since the prepared sample itself does not contain carbon, the detected trace amount of carbon is most likely due to organic contaminants adsorbed on the sample surface. This C 1s peak is critical for calibrating the XPS spectra, ensuring accurate measurement of the chemical states of the other elements. The high-resolution O 1s spectrum, shown in Figure 4c, reveals a distinct peak at 530.7 eV and a weak shoulder peak at 533.6 eV. This main peak corresponds to lattice oxygen (O1), indicating the presence of oxygen in the form of MoO_2_ crystals [36]. The weak peak position of this defect oxygen (O2) corresponds to the organic contaminants adsorbed or the defect in the MoO_2_ layer. The high-resolution Mo 3d XPS spectrum further elucidates the chemical composition and state of the Mo@MoO_2_ nanosheets, as shown in Figure 4d. This analysis reveals four distinct peaks, indicative of the presence of both metallic Mo and Mo in the Mo^4+^ oxidation state. The peaks at binding energies of 228 eV and 231.2 eV are attributed to Mo 3d_5/2_ and Mo 3d_3/2_, respectively, representing the metallic Mo state. Meanwhile, the peaks at slightly higher binding energies of 228.8 eV and 232.2 eV correspond to Mo^4+^ 3d_5/2_ and Mo^4+^ 3d_3/2_, respectively, indicative of the MoO_2_ phase [37,38]. The predominance of the peaks corresponding to metallic Mo over those for Mo^4+^ suggests that the nanosheets are mainly composed of Mo crystals, with a smaller proportion existing as MoO_2_ crystals. This observation aligns with the structural and compositional results from the SEM, TEM, and XRD analyses previously discussed. The coexistence of Mo and Mo^4+^ within the nanosheets enhance the electrochemical performance of supercapacitors through the synergistic effects of the different phases.

**Figure 5:**
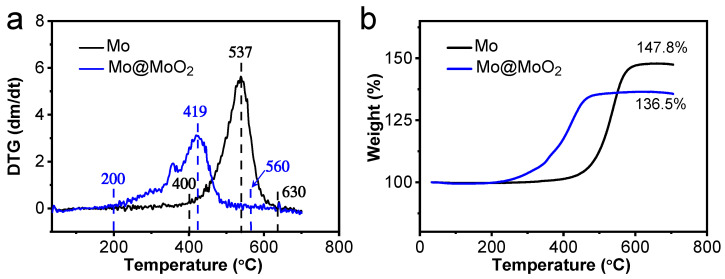
Thermogravimetric analysis of pure Mo powder and prepared Mo@MoO_2_ nanosheets. (**a**) Derivative thermogravimetric (DTG) curves. (**b**) Thermogravimetric analyzer (TGA) curves.

To further analyze the material composition of the Mo@MoO_2_ nanosheet composites, thermogravimetric analysis (TGA) tests were conducted in air atmosphere on both raw Mo powder and the synthesized Mo@MoO_2_ nanosheets from room temperature (~20 °C) to 700 °C at a heating rate of 10 °C min^−1^. The derivative thermogravimetric (DTG) curves depicted in Figure 5a illustrate that the pure Mo powder begins its oxidation process around 400 °C with a marked increase in mass change rate peaking at 537 °C. Beyond this peak, the rate of mass increase decelerates, eventually stabilizing at 147.8% of the initial mass at 630 °C. This indicates the complete oxidation of Mo to MoO_3_. In contrast, the Mo@MoO_2_ nanosheets exhibit the onset of oxidation at a significantly lower temperature (~200 °C), as seen in the blue line of Figure 5a. This earlier onset of oxidation is likely due to the outer MoO_2_ layer’s conversion to MoO_3_. The mass change rate for the nanosheets reaches its maximum at 419 °C, and by 560 °C the sample has fully transitioned to MoO_3_, with the mass stabilizing at 136.5% of the initial sample mass, as indicated by the blue line in Figure 5b. Further detailed analysis in Table S1 reveals that in the Mo@MoO_2_ nanosheet composite the mass percentage of MoO_2_ crystals, constituting the coating layer, is approximately 38.3%. Conversely, the mass percentage attributed to the Mo crystals, forming the core of the nanosheets, is about 61.7%. These results corroborate the core-shell composite structure of the Mo@MoO_2_ nanosheets, with metallic Mo forming the primary structure.

### 3.3. Electrochemical Properties

**Figure 6:**
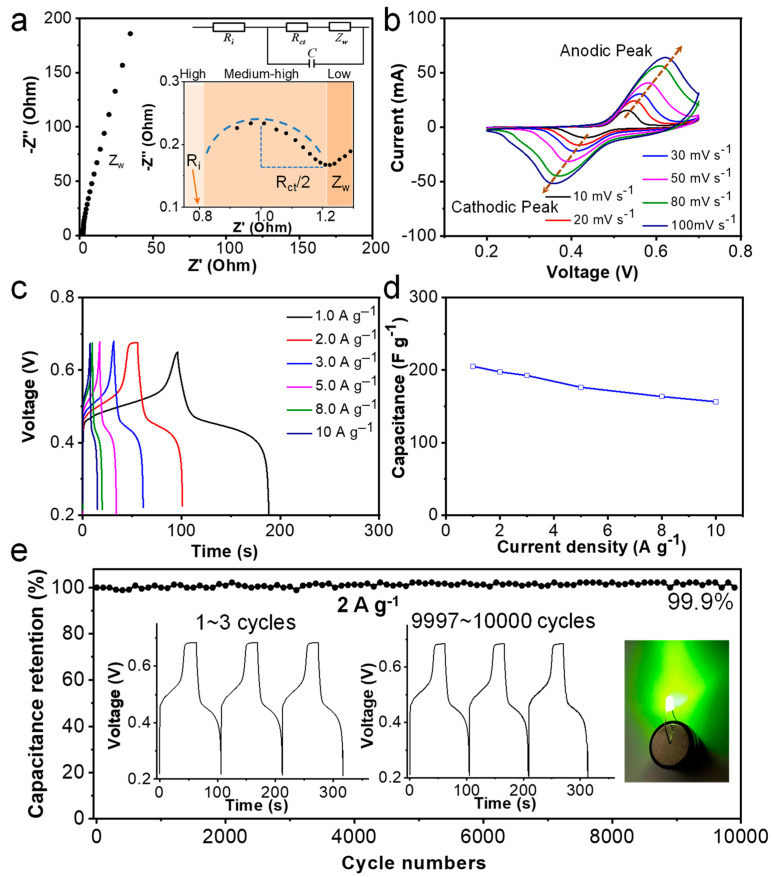
Electrochemical performance of the Mo@MoO_2_ nanosheets electrode. (**a**) Nyquist plots. Insets: the enlarged high-frequency and medium-high-frequency regions of Nyquist plots and the equivalent circuit model. (**b**) CV curves at different scanning rates from 10 to 100 mV s^−1^. (**c**) GCD curves at different current densities from 1 to 10 A g^−1^. (**d**) Rate capability curve with increasing current density. (**e**) Long cycling performance at a current density of 2.0 A g^−1^. Insets: the enlarged charge–discharge curves of the first three and the last three cycles, and an image showing the function of a green LED light powered by the assembled coin-type symmetrical supercapacitor.

The electrochemical energy storage capabilities of the Mo@MoO_2_ nanosheets were evaluated as electrodes for supercapacitors, utilizing a standard three-electrode system. This system comprised 2 mol L^−1^ KOH as the electrolyte, the Mo@MoO_2_ nanosheets as the working electrode, a platinum sheet as the counter electrode, and a saturated calomel electrode as the reference electrode. The electrochemical impedance spectroscopy (EIS) results shown in Figure 6a reveal the electrochemical behavior of the electrodes in three distinct parts: the interface resistance (*R*_i_) in the high-frequency area, the charge transfer resistance (*R*_ct_) in the medium-high-frequency area, and the Warburg impedance (*Z*_w_) in the low-frequency area. Specifically, the equivalent circuit model consists of elements such as *R*_i_, *R*_ct_, *Z*_w_, and capacitance (C), connected in series or parallel (Figure 6a inset) [39,40]. The *R*_i_, indicated by the semicircular start point’s intercept with the X-axis, relates primarily to the contact resistance between the electrode material, electrolyte, and current collector. The calculated *R*_i_ for the Mo@MoO_2_ nanosheets is exceptionally low at only 0.8 Ω, showing superior performance compared to many reported Mo-based electrode materials (see Table S2) [41,42,43,44]. This low resistance is attributed to the excellent electrical conductivity of the inner Mo layer (with a conductivity of up to 5.2 × 10^4^ Ω^−1^ cm^−1^) [45,46,47], which serves as an efficient conduit for the electrical contact between the Ni electrode substrate and the outer MoO_2_ material. The semicircle’s diameter in the EIS inset corresponds to the *R*_ct_, which influences the rate at which ions/electrons accumulate and are released on the electrode material’s surface. The Mo@MoO_2_ nanosheets’ *R*_ct_ was found to be remarkably low at 0.4 Ω, underscoring an enhanced performance over many existing Mo-based electrodes (see Table S2) [41,42,43,44]. This efficiency is likely due to the thin MoO_2_ layer on the surface, which ensures ample transport channels for ions/electrons at the Mo and MoO_2_ interface, thereby facilitating rapid energy storage. Additionally, the slope of the inclined curve in the EIS data signifies the *Z*_w_, which indicates the diffusion speed of ions/electrons within the electrode. A steeper slope denotes faster diffusion. The steep *Z*_w_ slope observed for the Mo@MoO_2_ nanosheets indicates rapid diffusion rates. This can be attributed to the homogeneous element-based Mo and MoO_2_ heterointerface, enhancing the transfer and diffusion of ions/electrons. Moreover, the monocrystalline nature of the prepared Mo and MoO_2_ contributes to superior electrical conductivity and ion transport capabilities, further bolstering the nanosheets’ electrochemical performance [48,49,50,51,52].

The cyclic voltammetry (CV) curves for the Mo@MoO_2_ nanosheets, presented in Figure 6b at different scanning rates from 10 to 100 mV s^−1^, demonstrate that the area under the CV curves increases with the increasing scanning rate. Moreover, due to the rapid scanning rate, some ions or electrons may not have sufficient time for insertion/deinsertion, resulting in a positive shift of the oxidation peak and a negative shift of the reduction peak. The slight shifts in the redox peaks, despite the increasing scan rates, suggest that the electrochemical reactions maintain their reversibility, a critical factor for the stability and efficiency of supercapacitors. The presence of two distinct redox peaks, around 0.4 V and 0.6 V, underscores the pseudocapacitive behavior attributable to the MoO_2_ surface layer of the electrode. These peaks represent the Faradaic reactions occurring at the surface of the MoO_2_, contributing to the overall capacitance through redox processes. The reversible redox reaction is as follows:

MoO_2_ + 4OH^−^ ↔ MoO_4_^2−^ + 2H_2_O +2e^−^ (3)

The inner metallic Mo layer within the Mo@MoO_2_ nanosheets, while not directly engaging in redox reactions, plays a critical role as a conductive pathway. This layer facilitates fast electron transfer throughout the material, thereby enhancing the overall electrochemical performance of the supercapacitor. Additionally, this metallic Mo layer contributes to the capacitance through electric double-layer formation, where charge storage occurs via ion adsorption without the need for chemical reactions. The cyclic voltammetry (CV) analysis further reveals that at various scanning rates, the redox peaks associated with the MoO_2_ layer are notably symmetrical and reversible. This symmetry and reversibility are indicative of the structural stability of the Mo@MoO_2_ nanosheets even under the stress of rapid charging and discharging cycles. Moreover, at different scanning rates, the two detected redox peaks are noticeably symmetrical and reversible indicating a stable structure for the Mo@MoO_2_ nanosheets, which alleviates the volume expansion of electrode materials and degradation of MoO_2_ pseudocapacitance during rapid charging and discharging.

The galvanostatic charge–discharge (GCD) tests of the Mo@MoO_2_ nanosheets (with a mass of 1.7 mg), performed at various current densities from 1 to 10 A g^−1^, offer critical insights into the nanosheets’ specific capacitance and rate capability. As illustrated in Figure 6c, the GCD curves exhibit notable symmetry, indicating that the electrochemical reaction has good reversibility. Meanwhile, there is indeed a small degree of polarization in the GCD curves, which can be attributed to the Ohmic polarization. This is caused by the contact resistance between the electrolyte and the electrode material, leading to a difference between the actual potential and the theoretical electrode potential. Additionally, a distinct voltage plateau between 0.4 to 0.6 V was observed, aligning with the redox peak positions observed in the cyclic voltammetry analysis (Figure 6b). This correlation confirms the pseudocapacitive storage mechanism attributed to the MoO_2_ layer, contributing significantly to the overall energy storage capacity of the nanosheets. The specific discharge capacities recorded for the Mo@MoO_2_ nanosheets are as follows: 205.1, 197.4, 192.4, 176.1, 163.3, and 156.3 F g^−1^ at current densities of 1, 2, 3, 5, 8, and 10 A g^−1^, respectively. These capacities indicate not only high specific capacitance but also excellent rate capability (Figure 6d), as evidenced by the ability to maintain a storage capacity retention rate above 76.2% even high current densities. The remarkable electrochemical performance of the Mo@MoO_2_ nanosheets is largely due to their core-shell structure. The inner metallic Mo layer plays a crucial role, with its high conductivity facilitating rapid ion/electron transfer and diffusion, thus speeding up the electrochemical reactions. Additionally, the outer MoO_2_ layer, with its abundant defects and active sites at the heterointerface between Mo and MoO_2_, further contributes to the enhanced storage capacity. The pseudocapacitive behavior of the MoO_2_ layer combined with the efficient electron transport provided by the metallic Mo core enable the Mo@MoO_2_ nanosheets to exhibit superior electrochemical properties.

The long-term cycling performance and stability of the Mo@MoO_2_ nanosheets as supercapacitor electrodes were evaluated through cyclic galvanostatic charge–discharge (GCD) tests at a current density of 2 A g^−1^ (Figure 6e). Impressively, after 10,000 cycles, the nanosheets showcased an outstanding specific capacitance retention rate of 99.9%. The GCD curves from the initial and final cycles, as shown in the inset of Figure 6e, exhibit remarkable overlap, further confirming the nanosheets’ excellent cyclic stability with no significant capacity loss. Beyond their robust cyclic performance, the Mo@MoO_2_ nanosheets were also integrated into a symmetrical coin-type supercapacitor device. This device (used in series of three) successfully powered a green LED light, drawing an output power of 0.06 W. This practical demonstration, as depicted in the inset of Figure 6e, underlines the Mo@MoO_2_ nanosheets’ capability to serve as effective energy storage materials in real-world applications. The superior energy storage performance of the Mo@MoO_2_ nanosheets is largely due to their vertical two-dimensional geometry and distinct core-shell architecture, which offer large specific surface area for electrolyte infiltration, numerous active sites for ion/electron storage, and enhance both the charge transfer speed and structural stability of the electrode material throughout repeated charge–discharge cycles. The ability to maintain high capacitance over thousands of cycles, coupled with their proven functionality in powering electronic devices, positions the Mo@MoO_2_ nanosheets as promising candidates for high-performance and durable supercapacitors.

## 4. Conclusions

This work introduces a novel one-step thermal evaporating vapor deposition (TEVD) technique to synthesize high-performance supercapacitor nanocomposite electrodes, culminating in the successful development of a non-carbon Mo@MoO_2_ core-shell heterostructure nanosheet on a Ni foam substrate. The Mo@MoO_2_ nanosheets, as supercapacitor electrode materials, have demonstrated exceptional electrochemical properties. These include extraordinarily low impedance values (*R*_i_ = 0.8 Ω and *R*_ct_ = 0.4 Ω), outstanding rate capabilities (with a storage capacity of 205.1 F g^−1^ at a current density of 1 A g^−1^ and maintaining a capacity retention rate of 76.2% even at an increased current density of 10 A g^−1^), and an unparalleled long-term cycling life and stability, exhibiting nearly unchanged capacity (~99.9% retention) after 10,000 charge–discharge cycles at 2 A g^−1^. The unique vertical two-dimensional geometric features and core-shell structure of the Mo@MoO_2_ nanosheets significantly enhance the charge storage capacity, accelerate charge transfer, and stabilize the electrode structure throughout repeated cycling. These characteristics are pivotal in achieving the remarkable rate performance and long cycling properties. Consequently, this study not only offers a high-performance, non-carbon-based electrode material for supercapacitors, but also extends the methodology for fabricating nanocomposite electrodes that leverage diverse energy storage mechanisms.

## Data Availability

The data that support the findings of this study are available from the corresponding author upon reasonable request.

## Supplementary Materials

The following supporting information can be downloaded at: https://www.mdpi.com/article/10.3390/nano14171404/s1, Figure S1: Schematic diagram of the developed TEVD system; Figure S2: EDS analysis of an individual Mo@MoO_2_ nanosheet; Table S1: TGA data analysis of Mo powder and Mo@MoO_2_ nanosheets, and the calculations of each component in the Mo@MoO_2_ nanosheets; Table S2: Comparison of the interface resistance (R_i_) and the charge transfer resistance (R_ct_) properties of different reported Mo-based supercapacitor electrode materials.

## References

[1] Wei T., Chen C., Chien H., Lu S., Hu C. (2010). A cost-effective supercapacitor material of ultrahigh specific capacitances: Spinel nickel cobaltite aerogels from an epoxide-driven sol-gel process. Adv. Mater..

[2] Salari H., Shayeh J. (2021). A unique 3D structured NiMoO_4_/MoO_3_ heterojunction for enhanced supercapacitor performance. Energy Fuels.

[3] Jacobson M.Z. (2009). Review of solutions to global warming, air pollution, and energy security. Energy Environ. Sci..

[4] Jacobson M.Z. (2020). Evaluation of nuclear power as a proposed solution to global warming, air pollution, and energy security. 100% Clean, Renewable Energy and Storage for Everything.

[5] Lu H., Tian K., Bu L., Huang X., Li X., Zhao Y., Wang F., Bai J., Gao L., Zhao J. (2021). Synergistic effect from coaxially integrated CNTs@MoS_2_/MoO_2_ composite enables fast and stable lithium storage. J. Energy Chem..

[6] Yi F., Ren H., Dai K., Wang X., Han Y., Wang K., Li K., Guan B., Wang J., Tang M. (2018). Solar thermal-driven capacitance enhancement of supercapacitors. Energy Environ. Sci..

[7] Zhao N., Fan H., Zhang M., Ma J., Du Z., Yan B., Li H., Jiang X. (2020). Simple electrodeposition of MoO_3_ film on carbon cloth for high-performance aqueous symmetric supercapacitors. Chem. Eng. J..

[8] Zhang X., Fu Q., Huang H., Wei L., Guo X. (2019). Silver-quantum-dot-modified MoO_3_ and MnO_2_ paper-like freestanding films for flexible solid-state asymmetric supercapacitors. Small.

[9] Lei D., Shang W., Zhang X., Li Y., Qiao S., Zhong Y., Deng X., Shi X., Zhang Q., Hao C. (2021). Facile synthesis of heterostructured MoS_2_-MoO_3_ nanosheets with active electrocatalytic sites for high-performance lithium-sulfur batteries. ACS Nano.

[10] Xu L., Zhou W., Chao S., Liang Y., Zhao X., Liu C., Xu J. (2022). Advanced oxygen-vacancy Ce-doped MoO_3_ ultrathin nanoflakes anode materials used as asymmetric supercapacitors with ultrahigh energy density. Adv. Energy Mater..

[11] Du W., Liu R., Jiang Y., Lu Q., Fan Y., Gao F. (2013). Facile synthesis of hollow Co_3_O_4_ boxes for high capacity supercapacitor. J. Power Sources.

[12] Zan G., Li S., Chen P., Dong K., Wu Q., Wu T. (2024). Mesoporous cubic nanocages assembled by coupled monolayers with 100% theoretical capacity and robust cycling. ACS Cent. Sci..

[13] Kong D., Lv W., Liu R., He Y., Wu D., Li F., Fu R., Yang Q., Kang F. (2023). Superstructured carbon materials: Design and energy applications. Energy Mater. Devices.

[14] Cheng A., Shen Y., Hong T., Zhan R., Chen E., Chen Z., Chen G., Liang M., Sun X., Wang D. (2022). Self-assembly vertical graphene-based MoO_3_ nanosheets for high performance supercapacitors. Nanomaterials.

[15] Zhai T., Wan L., Sun S., Chen Q., Sun J., Xia Q., Xia H. (2017). Phosphate ion functionalized Co_3_O_4_ ultrathin nanosheets with greatly improved surface reactivity for high performance pseudocapacitors. Adv. Mater..

[16] Wang L., Gao L., Wang J., Shen Y. (2019). MoO_3_ nanobelts for high-performance asymmetric supercapacitor. J. Mater. Sci..

[17] Li Z., Yu P., Zhong W., Zhang M., Li Z., Cheng A., Liang Y., Miao L., Yang X., Zhang H. (2021). Hydrothermal intercalation for the synthesis of novel three-dimensional hierarchically superstructured carbons composed of graphene-like ultrathin nanosheets. Carbon.

[18] Jiang Q., Kurra N., Alhabeb M., Gogotsi Y., Alshareef H.N. (2018). All pseudocapacitive MXene-RuO_2_ asymmetric supercapacitors. Adv. Energy Mater..

[19] Chang L., Chen S., Fei Y., Stacchiola D., Hu Y. (2023). Superstructured NiMoO_4_@CoMoO_4_ core-shell nanofibers for supercapacitors with ultrahigh areal capacitance. Proc. Natl. Acad. Sci. USA.

[20] Kumbhar V., Chodankar N., Lee K., Kim D. (2019). Insights into the interfacial nanostructuring of NiCo_2_S_4_ and their electrochemical activity for ultra-high capacity all-solid-state flexible asymmetric supercapacitors. J. Colloid Interface Sci..

[21] Xiao J., Wan L., Yang S., Xiao F., Wang S. (2014). Design hierarchical electrodes with highly conductive NiCo_2_S_4_ nanotube arrays grown on carbon fiber paper for high-performance pseudocapacitors. Nano Lett..

[22] Fu L., Qu Q., Holze R., Kondratiev V.V., Wu Y. (2019). Composites of metal oxides and intrinsically conducting polymers as supercapacitor electrode materials: The best of both worlds?. J. Mater. Chem. A.

[23] Kong D., Wang Y., Huang S., Hu J., Lim Y., Liu B., Fan S., Shi Y., Yang H. (2019). 3D self-branched zinc-cobalt Oxide@N-doped carbon hollow nanowall arrays for high-performance asymmetric supercapacitors and oxygen electrocatalysis. Energy Storage Mater..

[24] Qin J., Sari H.M., Wang X., Yang H., Zhang J., Li X. (2020). Controlled design of metal oxide-based (Mn^2+^/Nb^5+^) anodes for superior sodium-ion hybrid supercapacitors: Synergistic mechanisms of hybrid ion storage. Nano Energy.

[25] Liu J., Wu C., Gates I., Jia B., Huang Z., Ma T. (2023). Integrated electrode-electrolyte optimization to manufacture a real-life applicable aqueous supercapacitor with record-breaking lifespan. Energy Environ. Mater..

[26] Dai J., Yang C., Xu Y., Wang X., Yang S., Li D., Luo L., Xia L., Li J., Qi X. (2023). MoS_2_@Polyaniline for aqueous ammonium-ion supercapacitors. Adv. Mater..

[27] Ma B., Hao W., Ruan W., Yuan C., Wang Q., Teng F. (2022). Unveiling capacitive behaviors of MoO_2_ in different electrolytes and flexible MoO_2_-based asymmetric micro-supercapacitor. J. Energy Storage.

[28] Sharma M., Adalati R., Kumar A., Chawla V., Chandra R. (2021). Single step fabrication of nanostructured Cr_2_O_3_-MoO_2_ composite flexible electrode for top-notch asymmetric supercapacitor. Appl. Surf. Sci..

[29] Li X., Shao J., Li J., Zhang L., Qu Q., Zheng H. (2013). Ordered mesoporous MoO_2_ as a high-performance anode material for aqueous supercapacitors. J. Power Sources.

[30] Yang L., Sun W., Zhong Z., Liu J., Gao Q., Hu R., Zhu M. (2016). Hierarchical MoO_2_/N-doped carbon heteronanowires with high rate and improved long-term performance for lithium-ion batteries. J. Power Sources.

[31] Zhang L., Lin H., Zhai L., Nie M., Zhou J., Zhuo S. (2017). Enhanced supercapacitor performance based on 3D porous graphene with MoO_2_ nanoparticles. J. Mater. Res..

[32] Zhao C., Hu Y., Zhou Y., Li N., Ding Y., Guo J., Zhao C., Yang Y. (2021). Aerobic recovered carbon fiber support-based MoO_2_//MnO_2_ asymmetric supercapacitor with a widened voltage window. Energy Fuels.

[33] Ferrari A.G.-M., Pimlott J., Down M., Rowley-Neale S., Banks C. (2021). MoO_2_ nanowire electrochemically decorated graphene additively manufactured supercapacitor platforms. Adv. Energy Mater..

[34] Vaddiraju S., Chandrasekaran H., Sunkara M. (2003). Vapor phase synthesis of tungsten nanowires. J. Am. Chem. Soc..

[35] Zhou J., Deng S., Gong L., Ding Y., Chen J., Huang J., Chen J., Xu N., Wang Z. (2006). Growth of large-area aligned molybdenum nanowires by high temperature chemical vapor deposition: Synthesis, growth mechanism, and device application. J. Phys. Chem. B.

[36] Lin X., Yan P., Xu F., Wu W., Hu T., Wei C., Xu Q. (2019). Solid-phase synthesis of atomically thin two-dimensional non-layered MoO_2_ nanosheets for surface enhanced Raman spectroscopy. J. Mater. Chem. C.

[37] Smudde G., Stair P. (1994). The oxidation of Mo (100) studied by XPS and surface Raman spectroscopy: The onset of MoO_2_ formation and the formation of surface polymolybdate. Surf. Sci..

[38] Werfel F., Minni E. (1983). Photoemission study of the electronic structure of Mo and Mo oxides. J. Phys. C Solid State Phys..

[39] Haripriya M., Manimekala T., Dharmalingam G., Minakshi M., Sivasubramanian R. (2024). Asymmetric supercapacitors based on ZnCo_2_O_4_ nanohexagons and orange peel derived activated carbon electrodes. Chem. Asian J..

[40] Vasudevan S., Tharani S., Manickam M., Sivasubramanian R. (2024). A sol–gel derived LaCoO_3_ perovskite as an electrocatalyst for Al–air batteries. Dalton Trans..

[41] Shafi P., Dhanabal R., Chithambararaj A., Velmathi S., Bose A. (2017). α-MnO_2_/h-MoO_3_ hybrid material for high performance supercapacitor electrode and photocatalyst. ACS Sustain. Chem. Eng..

[42] Pham D., Patil R., Yang C., Yeh W., Liou Y., Ma Y. (2018). Impact of the crystal phase and 3d-valence conversion on the capacitive performance of one-dimensional MoO_2_, MoO_3_, and Magnéli-phase Mo_4_O_11_ nanorod-based pseudocapacitors. Nano Energy.

[43] Noh J., Yoon C., Kim Y., Jang J. (2017). High performance asymmetric supercapacitor twisted from carbon fiber/MnO_2_ and carbon fiber/MoO_3_. Carbon.

[44] Dai J., Qi X., Xia L., Xue Q., Luo L., Wang X., Yang C., Li D., Xie H., Cabot A. (2023). Aqueous ammonium-ion supercapacitors with unprecedented energy density and stability enabled by Oxygen vacancy-enriched MoO_3_@C. Adv. Funct. Mater..

[45] Worthing A. (1926). Physical properties of well seasoned molybdenum and tantalum as a function of temperature. Phys. Rev..

[46] Bardeen J. (1940). Electrical conductivity of metals. J. Appl. Phys..

[47] Shen Y., Deng S., Zhang Y., Liu F., Chen J., Xu N. (2012). Highly conductive vertically aligned molybdenum nanowalls and their field emission property. Nanoscale Res. Lett..

[48] Shen Y., Han Y., Zhan R., Zhao P., Zhang Y., Liu F., Chen J., She J., Xu N., Deng S. (2020). Study on pyramidal molybdenum nanostructures cold cathode with large-current properties based on self-assembly growth method. ACS Appl. Mater. Interfaces.

[49] Cho B., Park K., Baek J., Oh H., Lee Y.K., Sung M. (2014). Single-crystal Poly(3,4-ethylenedioxythiophene) nanowires with ultrahigh conductivity. Nano Lett..

[50] Day R., Bediako D., Rezaee M., Parent L., Skorupskii G., Arguilla M., Hendon C., Stassen I., Gianneschi N., Kim P. (2019). Single crystals of electrically conductive two-dimensional metal-organic frameworks: Structural and electrical transport properties. ACS Cent. Sci..

[51] Shen Y., Han Y., Zhan R., Chen X., Wen S., Huang W., Sun F., Wei Y., Chen H., Wu J. (2020). Pyramid-shaped single-crystalline nanostructure of molybdenum with excellent mechanical, electrical, and optical properties. ACS Appl. Mater. Interfaces.

[52] Cheng A., Shen Y., Cui T., Feng W., Zhan R., Pei Y., Tang S., Chen H., Deng S. (2023). Needle-shaped single-crystalline molybdenum micro-nano structure with high conductivity and excellent field emission properties: Implications for large-current cold-cathodes. ACS Appl. Nano Mater..

