# Supplementary material for: One-Step Synthesis of Heterostructured Mo@MoO2 Nanosheets for High-Performance Supercapacitors with Long Cycling Life and High Rate Capability
Source: Nanomaterials (Basel). 2024 Aug 28;14(17):1404. doi: 10.3390/nano14171404 (PMC11397695; doi:10.3390/nano14171404)
Supplement: Supplementary file 1 [file nanomaterials-14-01404-s001.zip › nanomaterials-3172987-supplementary.pdf]

Supporting Information

One-Step Synthesis of Heterostructured Mo@MoO$_2$ Nanosheets for High-Performance Supercapacitors with Long Cycling Life and High Rate Capability

*Ao Cheng, Yan Shen *, Tao Cui, Zhe Liu, Yu Lin, Runze Zhan, Shuai Tang, Yu Zhang, Huanjun Chen and Shaozhi Deng*

State Key Laboratory of Optoelectronic Materials and Technologies, Guangdong Province Key Laboratory of Display Material and Technology, School of Electronics and Information Technology, Sun Yat-sen University, Guangzhou 510275, China; chengao@mail2.sysu.edu.cn (A.C.); cuit7@mail2.sysu.edu.cn (T.C.); liuzh336@mail2.sysu.edu.cn (Z.L.); liny398@mail2.sysu.edu.cn (Y.L.); zhanrz3@mail.sysu.edu.cn (R.Z.); tangsh58@mail.sysu.edu.cn (S.T.); stszhyu@mail.sysu.edu.cn (Y.Z.); chenhj8@mail.sysu.edu.cn (H.C.); stsdsz@mail.sysu.edu.cn (S.D.)

* Correspondence: shenyan7@mail.sysu.edu.cn

The schematic diagram of the developed thermal evaporating vapor deposition (TEVD) system is shown in Figure S1. In a vacuum reaction chamber, a Mo boat as the evaporating source was inverted and secured on two copper electrodes. The Ni foam framework substrate was positioned directly below the Mo boat on a copper pillar platform. During the materials growth, the vacuum of chamber was pumped to a bottom pressure by a mechanical pump, thereafter Ar and H_2 gases were introduced into it from the top of the chamber. Then, the Mo boat was started heating by applying voltage to the two copper electrodes. An infrared thermometer was used to detect the temperature of the substrate and the Mo boat through a glass window.

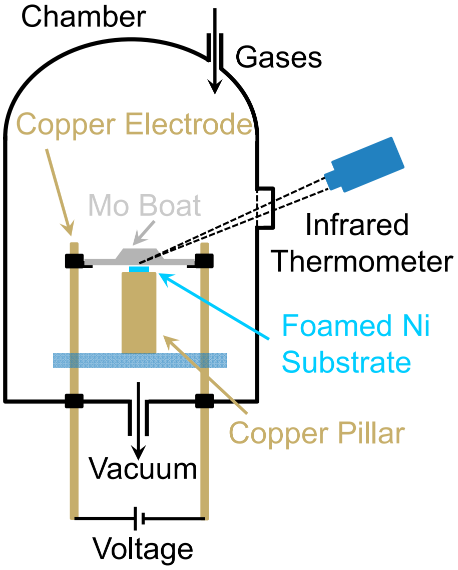

Figure S1. Schematic diagram of the developed TEVD system.

As shown in Figure S2, EDS mapping analysis of the Mo@MoO_2 nanosheets reveals distribution of Mo and O elements in the sample. The outline of the O distribution is larger than the region of the Mo (highlighted in Figure S2b), hence it can be inferred that O is mainly located on the surface of these prepared nanosheets. According to the EDS spectrum (Figure S2c), the atomic and mass percentages further confirm that metal Mo is the dominate part of the Mo@MoO_2 nanosheets while oxygen occupies a very little amount. The Cu signal comes from the TEM copper grid.

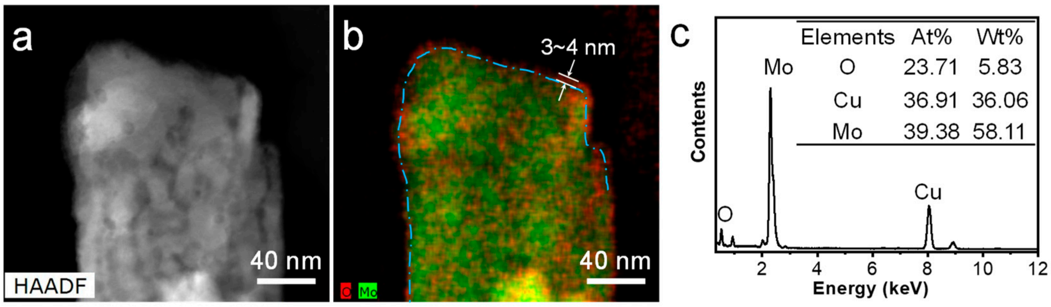

Figure S2. EDS analysis of an individual Mo@MoO_2 nanosheet. **(a)** High-angle annular dark-field (HAADF) image of the Mo@MoO_2 nanosheet. **(b)** EDS mappings of the Mo (green) and O (red) elements. **(c)** EDS spectrum of the Mo@MoO_2 nanosheets. Inset: the elements atomic and mass percentages of Mo, O, and Cu.

Table S1. TGA data analysis of Mo powder and Mo@MoO_2 nanosheets, and the calculations of each component in the Mo@MoO_2 nanosheets.

Sample	Initial Mass (mg)	MoO_3 (%)	Difference (%)	O in MoO_2 (mg)	MoO_2 (mg)	MoO_2 (%)	Mo (%)
Mo	5.305	147.8%	11.3%	0.498782	1.496346	38.3%	61.7%
Mo@MoO_2	4.414	136.5%					

Table S2. Comparison of the interface resistance (R_i) and the charge transfer resistance (R_{ct}) properties of different reported Mo-based supercapacitor electrode materials.

Electrode	Mo@MoO_2	MoO_3@C	MnO_2/MoO_3	Mo_4O_{11}	MoO_2	MoO_3	CF MnO_2/CF MoO_3
R_i (Ω)	0.8	~6	4.3	~35	~25	~45	25.4
R_{ct} (Ω)	0.4	~2	5.37	~20	~15	~50	79.9
Refs.	This work	[S1]	[S2]	[S3]	[S3]	[S3]	[S4]

References

[S1] J. Dai; X. Qi; L. Xia; Q. Xue; L. Luo; X. Wang; C. Yang; D. Li; H. Xie; A. Cabot; L. Dai; Y. Xu. Aqueous ammonium-ion supercapacitors with unprecedented energy density and stability enabled by oxygen vacancy-enriched MoO_3@C. *Adv. Funct. Mater.* **2023**, *33*, 2212440.

[S2] P. Shafi; R. Dhanabal; A. Chithambararaj; S. Velmathi; A. Bose. α-MnO_2/h-MoO_3 hybrid material for high performance supercapacitor electrode and photocatalyst. *ACS Sustainable Chem. Eng.* **2017**, *5*, 4757–4770.

[S3] D. Pham; R. Patil; C. Yang; W. Yeh; Y. Liou; Y. Ma. Impact of the crystal phase and 3d-valence conversion on the capacitive performance of one-dimensional MoO_2, MoO_3, and Magnéli-phase Mo_4O_{11} nanorod-based pseudocapacitors. *Nano Energy* **2018**, *47*, 105–114.

[S4]J. Noh; C. Yoon; Y. Kim; J. Jang. High performance asymmetric supercapacitor twisted from carbon fiber/MnO_2 and carbon fiber/MoO_3. *Carbon* **2017**, *116*, 470–478.
